# SENSORY LOSS IN AGING: SPOTLIGHTING JUNIOR INVESTIGATORS AND NEW DIRECTIONS IN THE SENSORY HEALTH INTEREST GROUP

**DOI:** 10.1093/geroni/igae098.1070

**Published:** 2024-12-31

**Authors:** Jennifer Deal, Jeannette Mahoney, Joshua Ehrlich

**Affiliations:** Chair; Co-Chair; Discussant

## Abstract

Our senses (hearing, vision, touch, taste, smell) are the means by which our brains perceive our environment, allowing us to engage and connect with our world. Recommendations for maintaining health and function with age include maintaining strong social connections and participating in intellectually stimulating mental and physical activities. However, our senses are what allow us to engage in these activities. Sensory loss is common in older adults, with over 2/3 of adults over age 55 experiencing loss in multiple senses. The goal of the Sensory Health Special Interest Group (SIG) is to foster collaboration and dissemination of research and clinical/educational resources for professionals interested in sensory health and the effects of sensory impairments on the overall health and well-being of older adults. This session will meet this goal by highlighting work that demonstrates the importance of sensory health with age conducted by outstanding Sensory Health SIG investigators who are early in their career path or new to the interest group leadership. As part of this symposium, we will discuss the impact of age-related hearing loss on functional brain connectivity, physical activity and disability. We will also discuss how olfaction is related to coronary heart disease and conclude with a discussion on multisensory integration and mobility. Sensory Health Interest Group Sponsored Symposium

